# REDuction of Antibiotic RESistance (REDARES) in urinary tract infections using treatments according to national clinical guidelines: study protocol for a pragmatic randomized controlled trial with a multimodal intervention in primary care

**DOI:** 10.1186/s12879-021-06660-0

**Published:** 2021-09-23

**Authors:** Ildikó Gágyor, Alexandra Greser, Peter Heuschmann, Viktoria Rücker, Andy Maun, Jutta Bleidorn, Christoph Heintze, Felix Jede, Tim Eckmanns, Anja Klingeberg, Anja Mentzel, Guido Schmiemann

**Affiliations:** 1 Department of General Practice, University Hospital Wuerzburg, Josef-Schneider-Str. 2, D7, 97080 Wuerzburg, Germany; 2 Institute for Clinical Epidemiology and Biometry (IKE-B), University of Wuerzburg, Wuerzburg, Germany; 3 Clinical Trial Centre Wuerzburg, University Hospital Wuerzburg, Wuerzburg, Germany; 4 Division of General Practice, Faculty of Medicine, Medical Center, University of Freiburg, Freiburg, Germany; 5 Department of General Practice, University Hospital Jena, Jena, Thuringia Germany; 6 Department of General Practice, Charité – Universitätsmedizin Berlin, corporate member of Freie Universität Berlin, Humboldt-Universität zu Berlin, and Berlin Institute of Health, Berlin, Germany; 7 Department of Health Services Research, Institute for Public Health and Nursing Research, University of Bremen, Bremen, Germany; 8 Robert-Koch-Institut, Berlin, Germany

**Keywords:** Antibiotic resistance, Urinary tract infections, Guideline adherence, Multimodal, Family physicians, Primary care

## Abstract

**Background:**

Urinary tract infections (UTIs) are a common cause of prescribing antibiotics in family medicine. In Germany, about 40% of UTI-related prescriptions are second-line antibiotics, which contributes to emerging resistance rates. To achieve a change in the prescribing behaviour among family physicians (FPs), this trial aims to implement the guideline recommendations in German family medicine.

**Methods/design:**

In a randomized controlled trial, a multimodal intervention will be developed and tested in family practices in four regions across Germany. The intervention will consist of three elements: information on guideline recommendations, information on regional resistance and feedback of prescribing behaviour for FPs on a quarterly basis. The effect of the intervention will be compared to usual practice. The primary endpoint is the absolute difference in the mean of prescribing rates of second-line antibiotics among the intervention and the control group after 12 months. To detect a 10% absolute difference in the prescribing rate after one year, with a significance level of 5% and a power of 86%, a sample size of 57 practices per group will be needed. Assuming a dropout rate of 10%, an overall number of 128 practices will be required. The accompanying process evaluation will provide information on feasibility and acceptance of the intervention.

**Discussion:**

If proven effective and feasible, the components of the intervention can improve adherence to antibiotic prescribing guidelines and contribute to antimicrobial stewardship in ambulatory care.

*Trial registration* DRKS, DRKS00020389, Registered 30 January 2020, https://www.drks.de/drks_web/navigate.do?navigationId=trial.HTML&TRIAL_ID=DRKS00020389.

## Background

Urinary tract infections (UTIs) are a frequent cause for patient visits in family medicine, with a prevalence ranging between 1.7 and 3.1% [1, 2]. Affected patients are usually treated with antibiotics as recommended by European guidelines [3]. Based on previous evidence, some guidelines recommend symptomatic treatment and delayed prescription of antibiotics for women with mild to moderate UTI symptoms who would prefer to avoid antibiotics. [4–7]. In the case of antibiotic treatment, guidelines recommend antibiotics with a narrow antimicrobial spectrum (first-line antibiotics), taking into account the local resistance situation [3]. Despite divergent recommendations, second-line antibiotics such as fluoroquinolones, still represent a large proportion of prescribed antibiotics for women with UTIs in Germany [2] and non-antibiotic treatments are rarely recommended [8]. Moreover, data on local resistance patterns of urine bacteria are not yet available for primary care physicians. The resulting inappropriate antibiotic use contributes to increasing resistance rates and weakens antibiotics as an important tool for serious or complicated infections [9, 10].

Implementation of clinical guidelines requires different strategies [11]. Several approaches to improve antibiotic prescribing by healthcare providers in primary care were explored, but none of them could be identified as the most appropriate strategy [12]. A recent Cochrane review investigating whether targeted clinician interventions influence antibiotic prescribing in acute respiratory tract infections found that various interventions such as shared decision-making, educational materials, educational meetings, audit and feedback, the use of point-of-care tests, etc. have impact on prescribing behaviour [13]. Multimodal interventions, such as educational programs with feedback about one’s own prescribing behaviour and the local resistance situation, have also shown positive effects on prescribing quality in ambulatory care [14, 15]. Components of multimodal interventions such as training of physicians in guideline-adherent treatments, audit and feedback to physicians [16], quality circles and prescribing feedback [17] for UTI, an electronic decision support system for medical practice [18] and regionally and nationwide benchmarking with other participating practices (social norm feedback) [19] for infectious diseases in general could improve prescribing quality in primary care. In addition, one study demonstrated a decrease in antimicrobial resistance of uropathogenic bacteria, when FPs followed the guidelines [20].

REDARES aims to implement the recommendations of guidelines in the management of UTIs in primary care, using practice-orientated information. It focuses specifically on the guideline-based selection, and not reduction, of those antibiotics—often required for UTI treatment—with the primary goal of improving the FPs’ guideline adherence and reducing the proportion of second-line antibiotics, defined as quinolones, cephalosporins, co-trimoxazole and others. To improve guideline adherence and to change prescribing behaviour of FPs, we plan to: (1) collect resistance data on uropathogens in ambulatory care in Germany and (2) provide practices with this data, combined with current guideline recommendations in an appropriate way. The primary hypothesis is that a multimodal intervention can improve the guideline adherence of FPs when prescribing antibiotics for UTIs, specifically in reducing the prescription rate of second-line antibiotics. The secondary hypotheses are that the number of antibiotics prescribed for patients with a UTI will decrease, and the guideline information will be used and accepted by the practice teams.

## Methods/design

REDARES is a randomized controlled trial (RCT) based on aggregated patient data with family practices being the unit of randomization. The accompanying process evaluation provides information on applicability and acceptance of the intervention. Furthermore, the users' perspective will inform the study team about the potential need to adapt information material and strategy.

### Population

The primary target population are FPs, working in general practices in four federal states in Germany: Baden-Wuerttemberg, Bavaria, Berlin-Brandenburg and Thuringia. Private practices are excluded. FPs’ practices will be block-wise randomized into intervention or control group. The randomization will be stratified per region and will be conducted with the software SAS Version 9.4.

### Intervention

We developed a multimodal intervention consisting of (a) information on guideline recommendations and on regional resistance data and (b) feedback of prescribing behaviour for FPs aiming to translate knowledge from practice guidelines and research into practice and to facilitate implementation of guideline adherent treatment [21]. Practices in the intervention group will receive data on local pathogens and resistance rates as well as key guideline information on the management of uncomplicated UTIs for FPs and patients, in print or electronic form. Furthermore, practices will obtain feedback on their individual antibiotic prescribing performance compared to other regional and supra-regional practices and advisory services via telephone quarterly. Thus the intervention will include both, passive components of educational intervention and active methods, which are more likely to induce change [21]. The individual components of the intervention will be developed, incorporating the findings of current process evaluations. The intervention will be evaluated throughout the study period to determine contextual factors necessary to achieve the outcomes and to identify the resources required to implement the intervention [22, 23].

### Outcomes

The primary outcome is the prescription rate of second-line antibiotics for UTIs at practice level after 12 months, based on aggregated data. We defined the prescription rate of second-line antibiotics per practices as the proportion of second-line antibiotics of all prescribed antibiotics for UTI. Second-line antibiotics are defined as antibiotics not recommended for treating uncomplicated urinary tract infections according to the national guideline (other than: Trimethoprim, Pivmecillinam, Nitrofurantoin, Fosfomycin or Nitroxolin). The primary hypothesis is, that the intervention will reduce the prescription rate of second-line antibiotics prescribed by 10%, from 43% to 33% after 12 months. Further hypotheses are that the number of antibiotics prescribed for patients with a UTI will decrease, and the guideline information will be used and accepted by the practice teams.

The study will provide data on efficacy of a physician-directed, multimodal intervention in family medicine, and its practical application to the daily routine. The accompanying process evaluation will provide information on the applicability and acceptance of the intervention and will inform the study team about potential adaptation of materials and strategy from the users` perspective.

Due to the systematic development and flexibility of the intervention, we expect that this will be amenable to practices’ standard care, and independent of the respective forms of organisation within the practice, such as individual workflow, roles and tasks delegated to physician assistants, and the information systems used. Beyond that, the results may represent a basis for the development of similar strategies in the implementation of recommended guidelines in different medical contexts. The knowledge gained through the collection of prescribing data in different patient management systems (PMS) contributes highly to the ongoing development of individual feedback systems that require regular and reliable validations.

### Study procedures

**Fig. 1:**
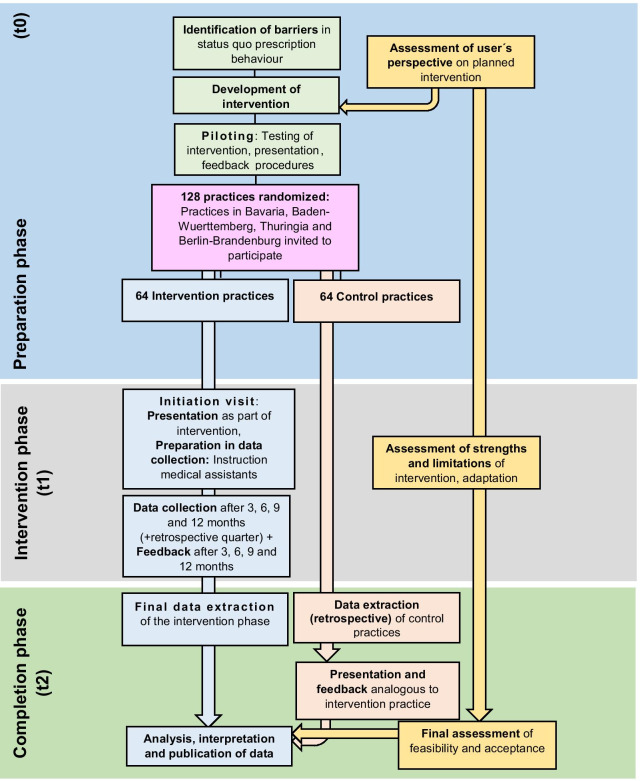
Flowchart of the REDARES study

The study consists of three phases, (1) the preparation phase (development), (2) the piloting phase and (3) the evaluation phase of the intervention (see also Fig. 1).

In the (1) preparation phase, the study team will develop components of the multimodal intervention (different guideline-orientated information for patients and FPs, information on regional resistance data, and regular feedback on physicians’ prescribing behaviour. (2) In order to adapt the study procedures to practice routines, the intervention was tested in a 6-month pilot phase before the start of the study in five non-participating practices. Adjustments based on individual feedback from the pilot practice teams were made as needed. The pilot phase did not affect the intervention itself, which will be the same in every practice. (3) The intervention and evaluation phase will be conducted in four regions in Germany as described above (see also population). After randomization, all intervention practices will be visited and provided with information on:

- The management of UTIs, including the current guideline recommendations, suggestions on how to use information for shared decision-making, information on resistance data (shown as a surveillance table), and several non-antimicrobial treatment approaches (delayed prescribing, painkillers, herbal remedies, etc.)
- How to deal with situations, such as differing patient preferences, guidance on treatment failure, and how to proceed with second-line antibiotics started by others

Intervention practices will receive individual feedback quarterly on their prescribing patterns, including benchmarking of all participating practices. They will have the opportunity to ask questions and receive oral feedback from a FP involved in the regional study team if they wish.

The effects observed in the intervention group will be compared to the control group (usual care). The control group will receive all intervention material after completion of the study. In the control practices, the physician assistants will then receive the training to obtain all data retrospectively. To reduce the interaction with the control practices and, thus, minimize potential bias, data sampling will be performed at the end of the study period in month 12 (see Fig. 1).

Process evaluation will start with FP interviews at the beginning of the intervention development process to assess what is locally feasible and acceptable. During intervention physician assistants and FPs from all intervention practices will complete a questionnaire about the feasibility of implementation in practice, generating important insight into this matter. Finally, after completion of the intervention phase, all practice teams will be asked to assess usability of the adapted intervention by a questionnaire in a group session with participating practices, results and final adaption of the intervention will be discussed.

### Data collection

Physician assistants will be trained to obtain treatment cases and data on prescribed antibiotics from the electronic patient record via a standardized training program to perform data sampling.

**Fig. 2:**
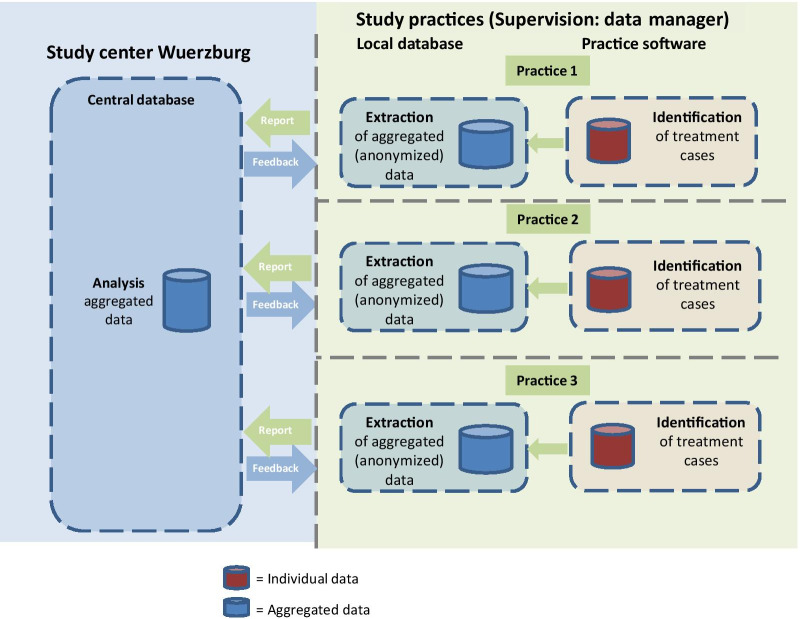
Data flow in REDARES

Data on UTI cases including number of UTI-related visits and antibiotics prescribed will be collected at practice level and transferred as aggregated data to the coordinating centre in Wuerzburg quarterly over a period of 12 months (see also Fig. 2). The final data extraction in the intervention practices will be performed 12 months after inclusion.

In addition, data will be collected in the intervention practices retrospectively for the first quarter of the year prior to study start. In control practices, data of the same five quarters will be collected at the end of the last study quarter (see also Fig. 1).

The number of UTI-related treatments, ICD-classifications and the number of consultations will be obtained quarterly. Additional data including sociodemographic data (age, insurance status, urine culture, pregnancy, allergies, history of recurrent UTI, pyelonephritis and referrals to specialists or clinics due to UTI) will be collected at the end of the study separately, when the number of cases will be high enough to be in line with the European General Data Protection Regulation [24]. This is necessary because German family practices are usually small sized with around 1000–1500 patients per FP.

We will also collect sociodemographic data of FPs, including their gender, age, duration of practice, practice characteristics, such as the practice situation, whether rural or urban, whether the practice is staffed by a single FP or multiple FPs and the number of patient encounters per year. This data will be collected in anonymized paper–pencil form and transferred to the coordinating center.

### Data analysis

Data analysis will follow the intention-to-treat principle. All randomized practices will remain in the allocated arm for analysis. All analyses are based on aggregated data obtained at practice level. For the primary analysis, we will use an analysis of covariance (ANCOVA) with an adjustment for the baseline rate of prescribing. Descriptive analysis of the frequency and prescription rate of second-line antibiotics, and the frequency of all antibiotics prescribed, the number of cases, and the dose and class of prescribed antibiotic will be assessed. In the secondary analysis, we will also adjust for size and region of the practice using ANCOVA. Furthermore, we will seek to categorize practices into two groups: those with a high rate of prescribing second-line antibiotics, versus those with a low rate. A comparison will be made between these two groups based on their demographics and general patient characteristics, for instance the age distribution of the practice, using Chi^2^-Test, t-Test or Mann–Whitney U test. To investigate the effect of demographics and patient characteristics of the practice, a multivariable logistic regression of the probability of a low rate of prescribing second-line antibiotics will be assessed. All secondary analyses will be explorative, with a significance level of 5%. SAS, R or SPSS software will be used to analyze the data.

### Sample size calculation

The primary endpoint will be the prescription rate of second-line antibiotics after 12 months. This will be measured at a practice level, and will be based on the aggregated data of each practice. A reduction in the rate of second-line antibiotic prescribing for UTIs is based on the following assumptions: in trials aiming to improve physicians’ guideline adherence, multimodal interventions such as training, focus groups and personal feedback, demonstrated a definite reduction in the prescribing rate of second-line antibiotics by more than 20% (absolute difference) [16, 17].

In comparison to former studies, REDARES aims to develop interventions without the need for expensive and time-consuming training or the provision of other educational media in participating practices. It is rather based on an information transfer and regular feedback, which, if proven effective, can be implemented in usual practice. Therefore, we would assume a conservative effect, in other words a 10% absolute reduction in the prescribing rate of second-line antibiotics. To adjust for differing baseline prescribing rates of second-line antibiotics, the sample-size calculation of the primary analysis was based on ANCOVA, with baseline prescribing rates as a covariate. Following Dicheva et al. 2015 [2] an assumption was made based on a quinolone prescribing rate of 43% of all antibiotics prescribed for women with a UTI. At the practice level, there is no data available for the standard deviation of prescribing rates. Therefore, we based our own experience on an assumed standard deviation of 20% and a moderate R^2^ of 0.25 of the covariate, equating to a correlation of 0.5 between the baseline and the prescribing rate after one year. To detect a 10% absolute difference in the prescribing rate after one year, between the control group (43%) and the intervention group (33%), a total sample size of 114 practices, 57 practices per group, are needed to achieve a power of 86% with a significance level of 5%. Assuming a dropout rate of 10% at the practice level, a sample size of 128 practices needs to be recruited.

### Patient and public involvement

Patient involvement in clinical trials has become increasingly relevant [25]. In a previous RCT, a close collaboration with a patient board was established to increase patient participation at different study levels [26]. Our research team is collaborating with a citizens’ forum consisting of ten participants, which has recently been established at the Department of General Practice, University of Wurzburg [27]. The forum was introduced to discuss the design of departmental scientific studies and ensure that they are comprehensible and relevant. The members of the forum were informed about the aims and processes of the study and will be asked for feedback. Furthermore, we will discuss the results and the ways how to disseminate the key messages.

The study advisory board consists of a FP, a pharmacist, a layperson and a scientist with experience in this field. They are involved in issues of practice feasibility and in the discussion of the results.

### Ethical considerations & dissemination of information

The Ethics Committee of the Medical Faculty, University of Wuerzburg, judged that the project did not involve any medical or epidemiological research on human subjects, and as such adopted a simplified assessment protocol. The project was approved without any reservation under the proposal number 20191106 01.

FP practice teams will obtain the treatment cases, and transfer the anonymized, aggregated data to the University of Wuerzburg. Feedback will be sent out in the form of aggregated data, such as the number of cases, and the antibiotic prescribed. There will be no transmission of individual patient data. Therefore, informed consent of individual patients was not needed. The data provided will be analyzed at the study center in Wuerzburg, which will provide a benchmark response. This procedure will guarantee anonymity.

## Discussion

We expect that the multimodal intervention that includes information on guideline recommendations, on regional resistance data and feedback of prescribing behaviour for FPs will enhance guideline adherence in this group. Continuous process evaluation will prepare for later implementation in practice if the intervention proves feasible.

A limitation of the study might be the manually extracted retrospective outcome data from the electronic patient records, which will rely on the documentation quality. Selection of research-interested practices is possible and the sample will probably not fully represent the usual practices.

We are sure the protocol of the study will be of interest for physicians, epidemiologists and researchers, who struggle with the increase of antibiotic resistance due to inappropriate antibiotic prescriptions, particularly as effective anti-infective agents are essential. If proven effective and feasible, the components of the intervention can improve adherence to antibiotic prescribing guidelines and contribute to antimicrobial stewardship in ambulatory care.

## Data Availability

The datasets used and/or analyzed during this study will be available from the corresponding author on reasonable request.

## Abbreviations

ANCOVA: Analysis of covariance
FPs: Family physicians
G-BA: Innovation Committee of the Federal Joint Committee
PMS: Patient management systems
RCT: Randomized controlled trial
REDARES: **RED**uction of **A**ntibiotic **RES**istance
UTIs: Urinary tract infections
